# Pushover Tests on Unreinforced Masonry Wallettes Retrofitted with an Innovative Coating: Experimental Study and Finite Element Modelling

**DOI:** 10.3390/ma14226815

**Published:** 2021-11-11

**Authors:** Jean-Patrick Plassiard, Mathieu Eymard, Ibrahim Alachek, Olivier Plé

**Affiliations:** 1 Laboratoire LOCIE, Campus Scientifique, Savoie Technolac, 73376 Le Bourget-du-Lac, France; olivier.ple@univ-smb.fr; 2 ANABAT Ingénieurs Conseils Sarl, Allée Paul-Cantonneau 5, 1762 Givisiez, Switzerland; m.eymard@anabat.ch; 3 AMOCER Group, Research & Development Department AMODIS, 69100 Villeurbanne, France; Ibrahim.alachek@amodis.eu

**Keywords:** masonry wallette, pushover test, strengthening, finite element modelling, damage mechanics, vertical loading mode

## Abstract

This paper investigates the mechanical contribution of an innovative coating applied on masonry wallettes compared to a traditional one. In both cases, the multifunctional coatings were insulating coatings intended for thermal refurbishment, but they could also be used to retrofit masonry. Uncoated specimens as well as coated ones were submitted to pushover tests to establish the strength gain. URM walls experienced brittle failures while the coated walls exhibited significant strength gains and strong ductility. The corresponding finite element models were developed. The behaviour of the URM walls was reproduced accurately in terms of strength and failure pattern. Models involving the coatings were used to partially retrieve the behaviour and to highlight the issues of a continuum approach.

## 1. Introduction

Human safety and achieving savings in energy are of the utmost importance for the current society. This general observation is pertinent for areas with buildings, in terms of their structural safety, the limitation of their carbon emissions, and the reduction of their energy consumption, among other factors. Concerning this last point, one current method to obtain a good level of thermal insulation consists in adding an external insulation layer, which is directly applied on walls. This solution is frequently encountered in refurbishment and also in new buildings because of its capacity to prevent thermal bridges [1]. Recently, a patented insulating coating based on silica aerogels, which are super-insulating materials, was developed in a French regional project called FUI Parex-IT (Institut Carnot 10/01/2015). It consists of a light mortar principally composed of a mineral binder and an insulating filler comprising granules of hydrophobic silica aerogel. This coating is here referred to as ISO. It is intended for the refurbishment of masonry walls involving hollow clay bricks. It has an apparent dry mass of approximately 300 kg/m^3^ and a thermal conductivity of 0.027 W/(m·K) [2]. From a mechanical point of view, the coating can enhance the stiffness and the strength of a masonry wall. In such cases, the coating has a multipurpose function. This coating could be even more useful for former buildings that may have been damaged over time and may not match the current standards anymore. As an example, old masonry walls were often made of dry vertical joints, and their filling with mortar is mandatory according to the current seismic standards [3]. Recently, an exploratory study showed that a coated masonry house may be compatible with medium seismicity, while this is not the case for the usual unreinforced masonry houses [4]. Furthermore, combined refurbishment solutions have been proposed [5,6], involving the application of both a thermal coating and a textile-reinforced mortar (TRM). As for studies that discuss the single mechanical reinforcement of masonry panels [7,8,9,10,11,12], the strength gain offered by these solutions can reach 500%. It is also worth noting that the ductility of masonry panels was found to be significantly increased when retrofitting materials were added to unreinforced masonry walls (URM). Recently, an innovative thermal-resistant geopolymer mortar was developed [13]. Masonry wallettes were built and then covered with this material or with other reinforcing materials. The diagonal shear tests performed on these samples showed that the shear resistances were similar and that the geopolymer mortar exhibited a higher ductility. In the current study, the objective was to identify whether the application of a sole coating could offer a convenient strength gain with varying mechanical characteristics. As these coatings are composed of inorganic materials, the fire resistance can be improved also [14]. For that reason, two different types of coatings were tested: the ISO coating presented above and a commercial finishing coating, which is here referred to as MGF. Shear walls made of hollow clay bricks were built for this study. Horizontal joints were made of a thin mortar layer. Then, each coating was applied on both sides of two walls, while two supplementary walls were left without any coating for comparison. As recommended in [3], these walls were submitted to pushover tests to define their respective lateral strengths. All the mechanical characteristics of the materials, the test configurations, and the results of the experiments are presented in Section 2. In particular, the evolution of cracks with the lateral loading was investigated using digital image correlation (DIC) techniques. Section 3 is devoted to the presentation of the finite element modelling of the investigated walls. An elastoplastic damage constitutive law was chosen to represent the behaviour of each wall’s component [15]. The simulations of the pushover tests are presented for the URM wall and showed good agreement with the experiments. However, the models incorporating coatings did not retrieve all the experimental features correctly, among them the shear strength and the ductility. As shown in Section 3.3, the modelling approach incorporating continuous finite elements can explain this difference. The Section 3.4 concerns the vertical loading of reinforced masonry and the phasing difference that was identified between the tests in the laboratory and the application on a real building. These aspects were investigated in terms of strength capacity and crack patterns. Finally, conclusions and outlooks for this work are given in Section 4.

## 2. Material Properties and Wall Experiments 

### 2.1. Material Properties

#### 2.1.1. Bricks

The unreinforced masonry wallette test specimens were built of hollow clay bricks. The clay bricks were referenced as GFR20 bricks by the manufacturer Wienerberger (Pont-de-Vaux, France) that was engaged for this project. Their dimensions were 500 mm (width) × 299 mm (height) × 200 (thickness). The GFR20 bricks from Wienerberger were rectified bricks with vertical perforations for distributed thermal insulation. We chose this thickness to be sure that the bearing capacity of the wall could be reached during the pushover test. The two bed-faces were resurfaced in order to be assembled with thin mortar joints and the brick geometry allowed the filling of the vertical joints. By comparing the bricks’ dry bulk density (620 kg/m^3^) and absolute density (1500 kg/m^3^), it was deduced that holes represent 59% of the apparent brick volume. The mechanical properties of the brick included the apparent brick volume. The manufacturer’s data showed an average compression strength of 8 MPa, perpendicularly to the bed joint. Moreover, measurement of the Young’s modulus was carried out by the authors during the compressive tests on the bricks using the digital image correlation (DIC) method. The software 7D [16] was used to estimate the Young’s modulus in the cells’ direction and also in the direction perpendicular to the cells. Average Young’s moduli of 1.55 GPa and 1.4 GPa were deduced in the direction perpendicular and parallel to the cells, respectively.

#### 2.1.2. Thin-Mortar Joints

An M10 class thin-mortar joint (Parexlanko, Saint-Pierre-de-Chandieu, France) was used to fill the horizontal bed joints. Its compressive strength was 14 MPa and its tensile strength was 4 MPa at 28 days, according to the manufacturer. The Young’s modulus was determined during the compression test by the authors. A value of 1115 MPa ± 356 MPa was derived from the three compression tests performed. 

#### 2.1.3. ISO and MGF Coatings

Three-point bending tests on 4 cm × 4 cm × 16 cm specimens were carried out to assess the coatings’ tensile strengths ($\sigma_{t}$) by flexion. Through compression tests on the remaining specimens, the compressive strength $\sigma_{c}$ and the Young’s modulus E for these coatings were obtained. A summary of all of the three-point bending tests and compressive tests is presented in Table 1. It is worth noting that the Young’s modulus of the ISO coating was far lower than those for the MGF coating and the bricks. The value of the Young’s modulus for the MGF coating was approximately one-fourth of the bricks’ modulus. Moreover, the compressive strength of the ISO coating was more than 20 times lower than that of the MGF coating and almost 40 times lower than that of the bricks. Assuming that the tensile behaviour of the coatings is linear elastic until the stress peak, the tensile strain $\varepsilon_{t}$ at the peak can be computed from the results in Table 1. Mean values of 1.75 × 10^−2^ and 4.3 × 10^−3^ were obtained for the ISO and MGF coatings, respectively. The deformable feature of the ISO coating was far more important than that of the MGF coating. Although the tensile strain of the bricks was not established during this study, it can be assumed that its value was also far less than that of the ISO coating.

### 2.2. Wall Specimens

Six specimens were constructed by professionals from the brick manufacturer to ensure correct mounting and to facilitate the repeatability of the results with regard to the masonry. The dimensions of each specimen were 1500 mm (width) × 1500 mm (height) × 200 mm (thickness). Table 2 regroups the different configurations tested, following a previous study [17]. It is worth noting that the vertical joints were kept dry in order to correspond to the masonry typology encountered before 1980. Masonry was built around a concrete beam in order to ensure the appropriate application of the boundary conditions during the experiments. The masonry was glued to the beam with the same mortar as described above. For the same purpose, a second beam was applied on top of the masonry wall.

**Figure 1:**
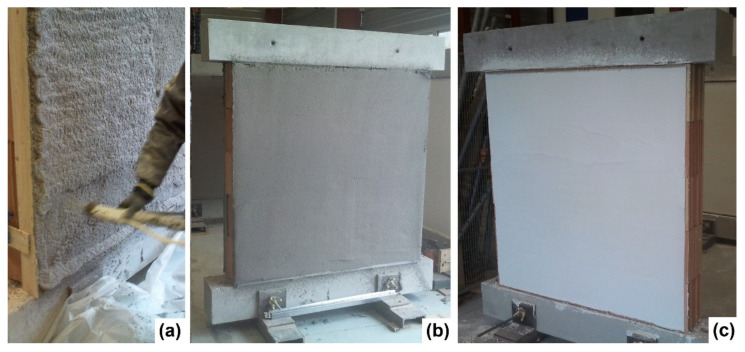
(**a**) Applying the second ISO layer on the masonry specimen; (**b**) masonry with ISO coating; (**c**) masonry with MGF coating.

Then, the coatings were sprayed on walls B3 to B7. The MGF coating consisted of one layer of 10 mm thickness, while the ISO coating was applied in two successive layers of 20 mm each to obtain a total thickness of 40 mm (Table 2). As the coatings covered only one side of the experimental walls, such a building method could have led to an out-of-plane response from the walls during the test. Hence, both sides of the walls were sprayed with the coating so that the symmetry condition was met during the test. The coated walls are presented in Figure 1.

### 2.3. Experimental Protocol

Assessment of the experimental lateral strength of masonry walls is usually carried out with shear cyclic tests [18,19,20,21] or diagonal tests [22,23,24], among others. Here, the pushover test was chosen, following the recommendations in [3]. In brief, a constant vertical load was applied on the wall first, and then a lateral displacement at a constant rate was imposed on the upper beam until the failure occurred. 

**Figure 2:**
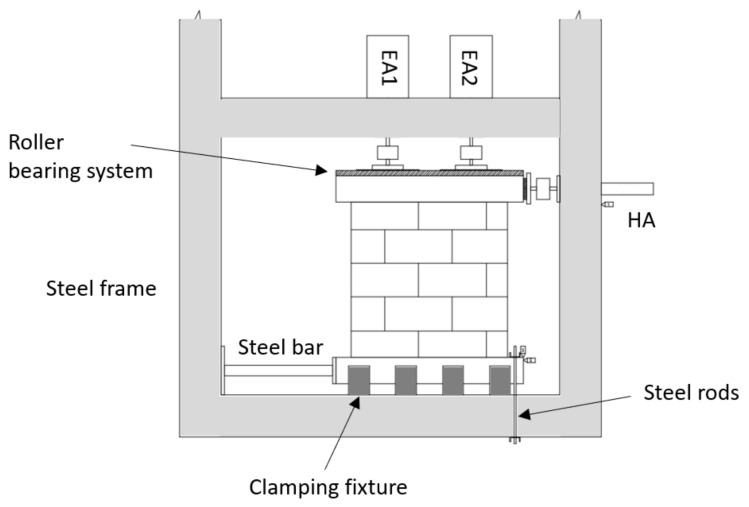
Experimental setup of a URM wall.

The tests were performed with a steel frame on which boundary conditions were applied. The beam inertia was equivalent to HEB400. Figure 2 depicts the URM wall with the boundary conditions acting on it during the test. The lower concrete beam of the wall was maintained against the frame by four vices blocking and centring the specimen inside the frame. This beam was also blocked laterally by a steel bar to prevent any sliding motion. Two tie rods, on the right, blocked the uplift of the beam during the horizontal loading. Two displacement transducers were used to assess the efficiency of the boundary conditions. For the upper concrete beam of the specimens, the horizontal (in-plane) displacement was allowed by a roller-bearing system. An UPN300 steel upper beam was used (with the roller-bearing system) to distribute the vertical load on the specimen. During the loading, the displacement fields of the front surface were computed in order to evaluate the appearance and propagation of cracks in relation to the loading. For this reason, a speckle with various grey levels was generated on this side of the walls. Digital images were obtained using a black and white numerical camera with a resolution of 16 million pixels. The image correlation software 7D [16] was used to assess the strain and displacement values of the specimen throughout the test. The crack patterns of both sides were systematically compared during the tests to verify that the behaviour was invariant in the out-of-plane direction. Tests showed a precision of 0.02 mm with the designated configuration. Thus, the level of precision was similar to that of typical displacement transducers; however, here, the displacement field was defined for the whole face of the wall and in both horizontal and vertical directions.

The pushover test is described in the following. The vertical load was applied by two actuators, while only one was required for the horizontal loading. Two electrical actuators (EA1 and EA2, with 120 kN capacity each) were used to apply the vertical pre-load, at a rate of 1 kN/s in 10 kN steps, until a 202 kN global load was reached. This pre-loading level was representative of the vertical load encountered in a three-story building, which corresponds to the building type for which this coating may be used. Furthermore, this loading value was close to the one used in [22], in which the authors observed shear failures of walls. As the two actuators were force-controlled, the 202 kN pre-loading setting was kept constant during the continuation of the test. Then, a horizontal in-plane displacement was applied by the hydraulic actuator (HA) to the lateral face of the upper beam. The horizontal displacement was imposed at a rate of 1 mm/min in steps of 1 mm until failure. All the actuators were used with ball joints so that no bending moment could be transferred to the wall.

### 2.4. Experimental Results

**Figure 3:**
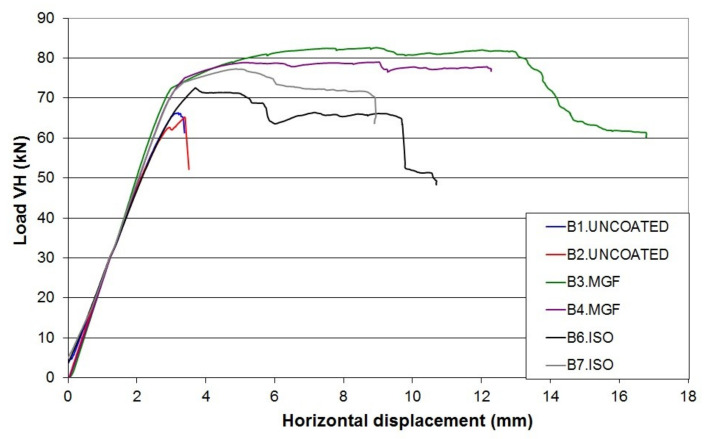
Force–displacement relationship of the tested specimens.

Figure 3 shows the force–displacement relationship for each tested specimen. The displacement corresponds to the lateral displacement of the beam near the contact with the hydraulic actuator. This displacement was derived from the DIC measurement, as the deformations of the metallic frame did not allow consideration of the displacement values from the actuator. The consistency of this measurement was verified by comparing the displacement values of one given point, as well as one value from a displacement transducer. 

First of all, the experimental results in the tests of the masonry response to the applied solicitations showed good reproducibility (Figure 3). The initial stiffnesses were measured in the linear part of the curves. The lateral stiffnesses were similar for the URM and the ISO walls, with values of about 22 kN/mm. The MGF wall appeared to be 3 kN/mm stiffer, with a value of about 25 kN/mm. Considering that the MGF coating was isotropic with a Poisson’s ratio of 0.2, a shear modulus G of 125 MPa ± 62.5 MPa was deduced from the Young’s modulus provided in Table 1. The supplementary stiffness $\Delta k_{s}$ provided by the coating was derived from the expression G×A/h, where A denotes the horizontal surface of the coating and h corresponds to the wall height. Considering the coatings of both sides, $\Delta k_{s}$ was equal to 2.5 kN/mm ± 1.25 kN/mm, which was quite consistent with the experimental value indicated above. The URM walls (B1 and B2) underwent brittle failures with an average maximum force of around 65 kN and an average maximum displacement at failure of around 3.5 mm. In comparison, the ISO-coated wall specimens (B6 and B7) showed more ductile behaviour, with an average maximum force of 75 kN and an average maximum displacement at failure of around 9 mm. This was even more noticeable for the MGF walls (B3 and B4), which exhibited a maximal strength of about 90 kN. It is safe to assume that the change of slope, observed between 3 mm and 4 mm for the coated specimens, corresponded to the propagation of the failure inside the masonry. Thus, this coating, applied here on the two sides of the specimens, allowed a 14% increase in the maximum horizontal force. Moreover, horizontal displacements at failure were almost three times greater, which could be useful in terms of energy dissipation during a seismic event.

**Figure 4:**
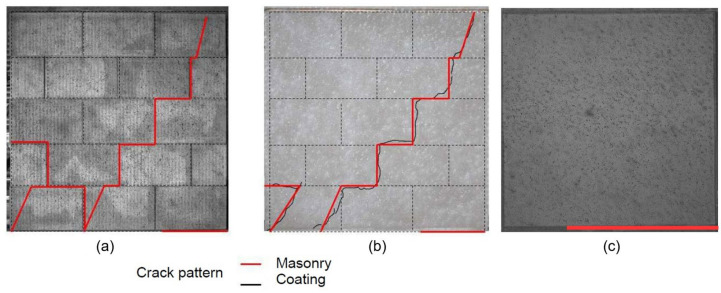
Crack patterns on the wall surfaces at masonry failure for specimens: (**a**) URM B1, (**b**) ISO-coated B7, and (**c**) MGF-coated B3.

In terms of the failure mode, similar failure patterns were also observed. Figure 4 shows examples of the crack patterns for a URM wall (B1) and an ISO-coated specimen (B7). These two types of specimen experienced stair-shaped shear failures with crack propagation along the horizontal and vertical joints of the masonry. This feature was noticed after manually removing the ISO coating from the masonry panel at the end of the test. It is also important to note that, along this crack pattern, bricks in contact with a concrete beam showed a diagonal crack, while this type of failure did not occur for inner rows of bricks. The flexural failure was the predominant failure mechanism for both specimens involving the MGF coating. The relevant crack was characterised by a tension failure in the lower bed joint (Figure 4c). No debonding of the coating was observed, even after the masonry failure, for either type of coating.

**Figure 5:**
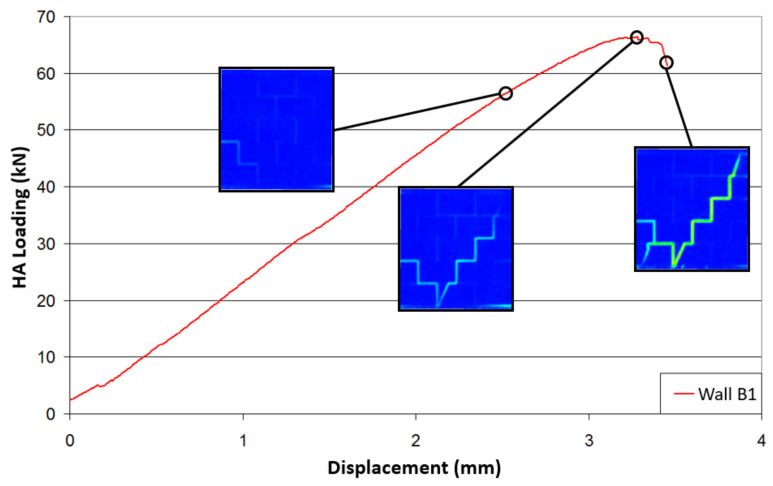
Evolution of cracking during the test of URM wall B1.

**Figure 6:**
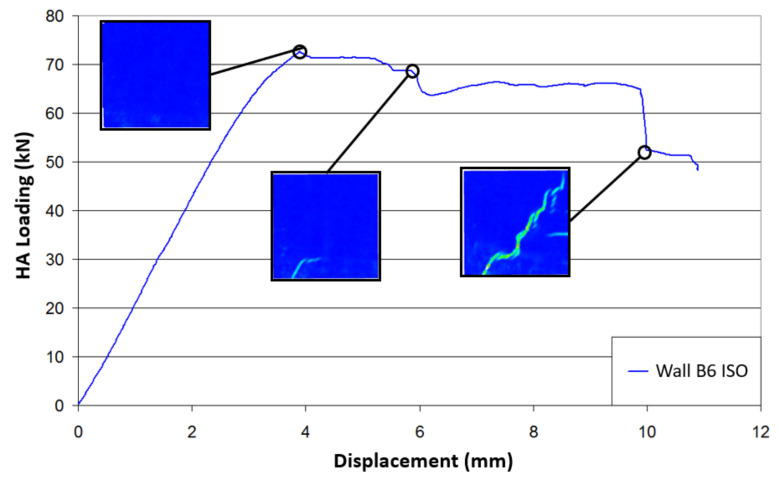
Evolution of cracking during the test of ISO-coated wall B6.

Closer analysis of the crack pattern evolution in the different specimens was undertaken; for example, by using the shear deformation $\varepsilon_{shear}$ displayed by 7D and calculated from the principal deformation (Equation (1)). These deformations enabled good observations of the failure lines, regardless of their orientation. The equation for calculating the shear deformation is as follows:

(1) $$\varepsilon_{shear}=\frac{\varepsilon_{max}-\varepsilon_{min}}{2}$$

where $\varepsilon_{max}$ and $\varepsilon_{min}$ denote the major and minor principal strains, respectively. To investigate the behaviour of the specimen, a qualitative study was carried out first. Indeed, once a crack opens, the measured strain may no longer be pertinent, but the appearance and opening of cracks are easy to identify with this variable. Figure 5 and Figure 6 show the evolution of the crack pattern along the effort–displacement curve for the specimens B1 and B6, respectively. For the wall B1, the stair-shaped shear failure did not develop first. Indeed, small cracks due to masonry faults could already be noticed in the left bottom edge during the pre-loading phase. At a load of 57 kN, theses cracks were still relevant, but the initiation of a flexural crack at the opposite side was also noticed. At the maximal load, the stair-shaped shear failure occurred, but the flexural failure mode was still present. At this point, the principal strains computed by the DIC analysis indicated that the horizontal joints involved in the cracks underwent shear strains, while tensile strains were noticed along the vertical joints. The post-peak behaviour was rather brittle. Finally, the relevant failure mode was the stair-shaped one, but it seems that the test configuration almost corresponded to the configuration of the transition between a flexural failure and a shear failure. Figure 6 shows the crack pattern on the external surface of the coating for the wall B6. Here, the measured strains could not be related to those of the masonry. It can be seen that the outside of the ISO coating did not crack before the loading peak was reached. From a thermal point of view, this means that the coating would be able to provide continuous insulation until the failure of the masonry occurs. It means that the critical aspect is the mechanical behaviour. Here, the ductility was far more important than for the URM wall. It can be observed that the shear strength decreased by 10% only when the displacement was twice that at the peak. Furthermore, the corresponding strength was more or less the same as the URM specimens at the peak. The crack patterns in Figure 5 and Figure 6 were similar at failure. The coating did not modify the shapes of the cracks or the failure mode, but its deformation ability seemed to postpone the failure propagation.

The experimental increase in strength $\Delta F_{exp}$ for the ISO walls compared to the URM walls was about 10 kN (Figure 3). As the crack patterns were very similar for both types of wall, this increase can be attributed to the ISO coating. DIC analysis of the URM walls showed the presence of tensile strains along the vertical joints and the development of cracks in the bricks, while shear strains occurred in the horizontal joints. This means that the ISO coating was subject to tensile stress along those vertical joints and also along the brick cracks. The strength gain ΔF was also estimated analytically when the crack had completely developed through the wall. In order to compute this gain, three assumptions were required:

- The shear stresses along the horizontal joint could be neglected in a first approach;
- The cracks generated in the coating thickness would be negligible compared to the coating thickness;
- Along the vertical joints and the brick cracks at failure, the coating would be subject to a constant tensile stress equal to its tensile strength $R_{t}$ over its full height, and the tensile behaviour of the coating would be elastoplastically rigid [23].

With these assumptions, the analytical strength gain $\Delta F_{computed}$ could be evaluated as follows (Equation (2)):

(2) $$\Delta F_{computed}=2\cdot e\cdot h\cdot R_{t}$$

where *e* and *h* denote the coating thickness on each side of the wall and the height of the wall, respectively. Using the experimental values of these parameters, $\Delta F_{computed}$ was equal to 8.4 kN, which was slightly lower than the increase of 10 kN obtained experimentally. It is worth noting that this relation was not valid for the walls with the MGF coating. Further investigation is still required with regard to the coating ductility and its behaviour under cracking modes 1 and 2. Nevertheless, the use of a coating, even with moderate mechanical properties, may represent a good solution to improve the strength and the ductility of URM walls. 

## 3. Numerical Modelling

### 3.1. Presentation of the Numerical Approach

Several numerical methods can be used for the modelling of masonry structures, depending on the scale of study and the knowledge of the mechanical properties. They can be grouped in two families: macro-modelling and micro-modelling approaches [24]. The first consider the brick, mortar and their interfaces as homogenised in a single equivalent material. These methods were not completely suitable for the current study as the objective was to retrieve the different possible failure modes. The second family includes the simplified and the detailed micro-modelling approaches. In the first, the bricks are resized in order to be in contact and the real mortar joints and interfaces are replaced with an interface element. On the other hand, the detailed micro-modelling approach considers the real sizes of brick units and mortar joints. They are modelled as continuous elements, with discontinuity being possible with the insertion of interface elements that are used between them. 

In the current study, finite element modelling was used to simulate the behaviour of the different masonry walls. Some of the materials’ characteristics were not evaluated during the experiment, making it difficult to define all of the numerical parameters. Therefore, the main purpose in this part of the study was to calibrate the model for the URM wall and apply it to the two types of coated walls, for which the main parameters were known. The nonlinear approach based on micro-modelling was retained [24], but the interface elements that usually represent the mortar unit were replaced with continuum elements. Aside from addressing the issue of modelling the coexistent mortar joints and coating, this choice offered the possibility of using the same constitutive law for all of the materials involved. The ability of constitutive models, such as the concrete damage plasticity model, to simulate the behaviour of reinforcing materials has been demonstrated [25,26,27]. Here, an elastoplastic model that also incorporated damage was adopted [15]. Moreover, this model, initially developed for concrete modelling, includes hysteretic behaviour and crack reclosure, among other behaviours noticed in masonry materials. A short presentation of the constitutive law is provided below, but a detailed review of its theoretical background can be found in [28]. 

The damage approach requires the identification of the relation between the effective and the total stresses. The effective stress tensor ${\tilde{\sigma }}_{kl}$ is used to calculate the total stress tensor $\sigma_{ij}$ (Equation (3)). The corresponding tensor involves a positive part ${\tilde{\sigma }}_{kl}^{+}$ for the tensile stresses and a negative part ${\tilde{\sigma }}_{kl}^{-}$ for the compression stresses. Four damages types are considered. The first is isotropic pre-peak damage under tension, which is denoted $D_{0}^{t}$. If the pre-peak behaviour is not linearly elastic, this pre-peak damage is not null. Beyond the peak, the orthotropic damage under tension $D^{t}$ is considered, which corresponds to the localization of the tensile crack. The value of $D^{t}$ is driven by the fracture energy under tension $G_{FT}$. Mesh dependency in the results is avoided by considering the finite elements’ size in the regularization during the fracturing process [29]. The shear-compression damage $D^{S}$ is also incorporated. It is isotropic and related to the plastic dilatancy and the characteristic dilatancy threshold $\varepsilon_{k,s}$. The smaller the value of $\varepsilon_{k,s}$, the more ductile the behaviour obtained is. Finally, compression stress applied to an existing crack involves the reclosure of tensile cracks. For this reason, a damage variable $D^{r}$ is considered also. Its value is derived from the corresponding compression stress value $R_{R}$ and from the energy $G_{FR}$ necessary for the crack reclosure.

(3) $$\sigma_{ij}=\left(1-D^{S}\right)\left[\left(1-D_{0}^{t}\right){\left(1-D^{t}\right)}_{\mathrm{ijkl}\;}{\tilde{\sigma }}_{kl}^{+}+{\left(1-D^{r}\right)}_{\mathrm{ijkl}}{\tilde{\sigma }}_{kl}^{-}\right]$$

The elastic behaviour of the effective stresses is determined by Hooke’s law. Let us consider the tensor of elasticity C, with C being a function of the Young’s modulus E and the Poisson ratio υ. Then, the effective stress tensor ${\tilde{\sigma }}_{kl}$ can be written as a function of the elastic strains $\varepsilon_{kl}^{e}$ (Equation (4)):

(4) $${\tilde{\sigma }}_{ij}=C_{ijkl}\;\varepsilon_{kl}^{e}$$

Two plastic criteria are incorporated for the description of the plasticity. Inelastic strains enter when the limit of a Rankine criterion is reached. This threshold corresponds to the uniaxial tensile strength $R_{T}$, after which inelastic strains develop in the direction of the major tensile stress. The compression shear-plasticity is monitored by a Drucker Prager criterion $f^{DP}$, for which two other parameters are mandatory: the uniaxial compression strength $R_{C}$ of the material and the Drucker Prager confinement coefficient δ. This last is computed from Equation (5) and depends on the internal friction angle ϕ.

(5) $$\delta =\frac{2\sqrt{3}\sin\phi }{3-\sin\phi }$$

The plastic flow in the shear behaviour is non-associated. Studies on concrete have indicated that it is adequately controlled by the dilatancy coefficient β and by the characteristic strain $\varepsilon_{k,s}$ indicated above. The hydration advancement coefficient ζ is also discussed in the Section 3.4, where the effect of the in-situ stress is considered. This parameter ranges from 0.0 to 1.0, corresponding to non-solidified and completely solidified behaviour, respectively. It was set automatically to 1.0 in the following, except for the simulations discussed in Section 3.4.

**Figure 7:**
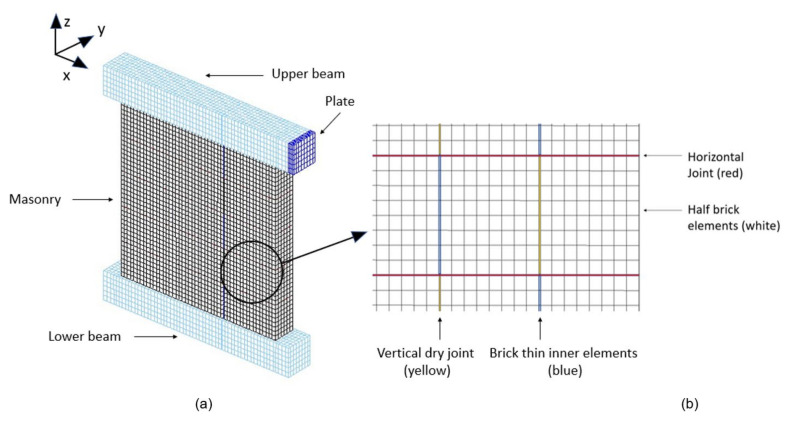
Numerical configuration for the URM wall (**a**) and front view of the masonry mesh (**b**). This figure was published in Computers and Structures, Vol 254, Jean-Patrick Plassiard, Ibrahim Alachek, Olivier Plé, Damage-based finite-element modelling of in-plane loaded masonry walls repaired with FRCM, Page, Copyright Elsevier (2021) [30].

**Figure 8:**
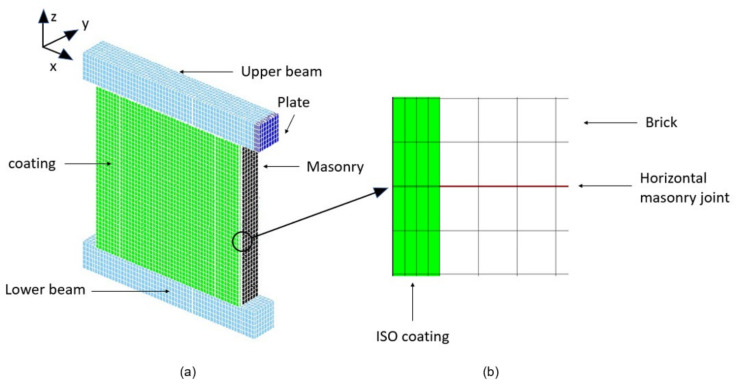
Numerical configuration of the MGF wall (**a**) and side view of the coating model (**b**).

All materials of the masonry units and of the coatings were modelled with the previously described constitutive law, except to the thin inner elements of the masonry. Furthermore, Hooke’s law was considered in order to avoid the premature cracks of those elements and the resulting unrepresentative failure of the masonry [30]. The Young’s modulus and the Poisson’s ratio of the other brick elements were also assigned for the thin elements. The concrete beam and the steel plates were modelled as linearly elastic, with Young’s moduli equal to 30 GPa and 210 GPa, respectively, and the same Poisson’s ratio of 0.2. The global presentation of the mesh for the URM wall is depicted in Figure 7, along with a detailed view of the masonry units. The model of the ISO-coated wall is presented in Figure 8. For both ISO and MGF walls, each coating was represented with four elements of thickness. For the thicknesses of the ISO and the MGF coatings, each element layer had a thickness of 1 cm and 0.25 cm, respectively. These elements were contiguous with those of the masonry on each side of the wall. In accordance with the experiments, a spacing of one element was left without a coating element at the bottom and the top of the masonry to avoid any contact between the coating and the concrete beams. Even though the interface characteristics between the coating and the masonry could be defined, as in [23], it was assumed that each coating type could be represented as a single material. Indeed, no debonding areas between the masonry and the coatings were noticed following the experiments. Therefore, perfect adhesion was assumed, as for previous models of FRP and TRM strips on masonry and of beams units [31,32,33,34]. Finally, the model shown in Figure 7 [30] was represented with 20,868 finite elements, while the model of the coated wall involved 41,932 elements (Figure 8).

The pushover tests were simulated in accordance with the experiments. The bottom surface of the lower beam was fixed in the three directions. In order to generate a similar load to that in the experiment, both load and displacement controls were required. During the first phase of loading, a vertical stress was applied progressively on the top surface of the upper beam until the target load of 202 kN was reached. Then, in the second phase, a progressive displacement along the x-axis was imposed on the middle point of the right surface of the plate, while the vertical loading was maintained (Figure 8). Here, corresponding displacement increments of 3 × 10^−2^ mm were applied at each step, as in a previous study [30].

### 3.2. Calibration and Simulation Results

Table 3 presents the set of parameters that were assigned to the masonry components and to the two coatings. The parameters for the masonry were first calibrated for the URM wall, so that the behaviour of the URM masonry could be calibrated in terms of the load–displacement and crack pattern evolutions. The Young’s modulus of the bricks was calibrated to reproduce the initial stiffness of the URM wall, while those of the bed joints and horizontal joints were fixed in a second step to reproduce the appearance of the mortar cracks. The compression strength $R_{C}$ of the brick was set to obtain a failure by corner crush. Then, the strain at the tension peak $\varepsilon_{PT}$, the fracture energy under tension $G_{FT}$, the strain at the compression peak $\varepsilon_{PC}$, the reclosure characteristic stress $R_{R},$ and the crack reclosure energy $G_{FR}$ were fixed to the values recommended in [15]. The characteristic strains $\varepsilon_{k;s}$, dilatancy coefficient β, and Drucker Prager coefficient δ were set in accordance with the values used in [30] as far as possible. In the second step, the parameters of the coated walls were defined. Several parameters were deduced from the experiments, such as the compression and tensile strengths of both coatings. The Young’s modulus of the ISO coating was also set to the value derived from the characterization tests while that of the MGF coating was calibrated to reproduce the experimental value for the initial stiffness of 25 kN/mm.

**Figure 9:**
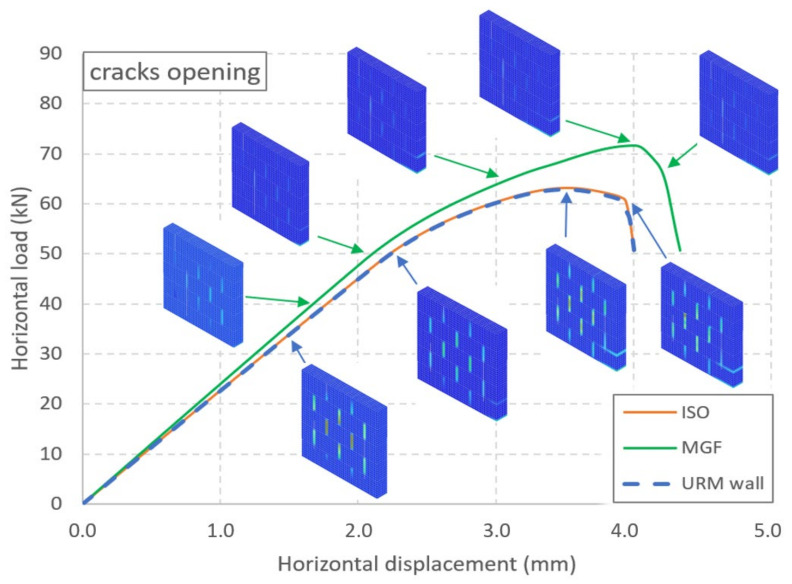
Numerical force vs. displacement curves for the three types of wall and the evolution of the opening of the cracks for the URM and MGF walls.

The force–displacement curves for the three types of wall are presented in Figure 9. For the URM wall, a linear trend up to 50 kN is noticeable (Figure 5). Then, a curve emerges until the maximal strength is reached at a horizontal displacement of 3.52 mm and a horizontal load of 62.8 kN. The strength decreases rapidly after the peak is reached. Thus, the results for the URM wall were similar to the experimental ones. On the other hand, the models of the coated walls failed to reproduce the experiments adequately. The model of the ISO wall exhibited almost the same evolution as the URM wall. A maximal strength of 63.2 kN was reached for a displacement equal to 3.5 mm. This represented a gain of less than 1%, as if the coating had no influence. Moreover, the ductile behaviour was also not reproduced. Similar findings were obtained for the MGF wall. The maximal strength was 71.73 kN, reached at a displacement of 4 mm. Here, the coating had an influence, but this was still less than the maximal value of around 90 kN from the experiments. The strength dropped quickly after the peak, as for the two other wall models. As an intermediate conclusion, we found that two major aspects were not reproduced adequately by the simulations: the maximal strength and the ductility.

**Figure 10:**
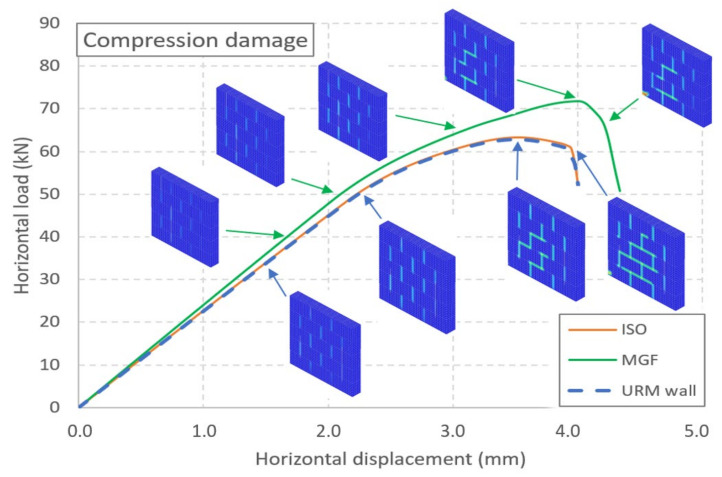
Numerical force vs. displacement curves for the three types of wall and the evolution of the damage under compression for the URM and MGF walls.

The crack patterns and the compression damage in the masonry are presented for several loading levels of the URM and MGF walls in Figure 9 and Figure 10, respectively. Here, we focused on the pattern of these two quantities and not on their value. Up to around 50 kN, cracks were concentrated in the dry vertical joints (Figure 9). Then, a crack developed along the base joint below the right brick of the first row, which could have been linked to the start of the nonlinearity of the curve. For higher values, a second crack even appeared in the joint located between the first and second rows. The crack pattern did not evolve after that, even once the maximal strength was reached. The evolution of the compression damage (Figure 10) indicated that the damage was located in the vertical joints for most of the rising portion of the curve. Then, for both types of walls, a staircase pattern developed just before the maximal strength was reached. Finally, the strength decreased with the crushing of the corner brick. Cracking of joints propagated across the major part of the masonry, but this can be linked to the instability caused by the crushing of the brick. As a conclusion, we found that the pattern evolution of the damage under compression seemed to be a valuable index for the estimation of the state of masonry walls subject to staircase failure.

### 3.3. Limitations of the Model

**Figure 11:**
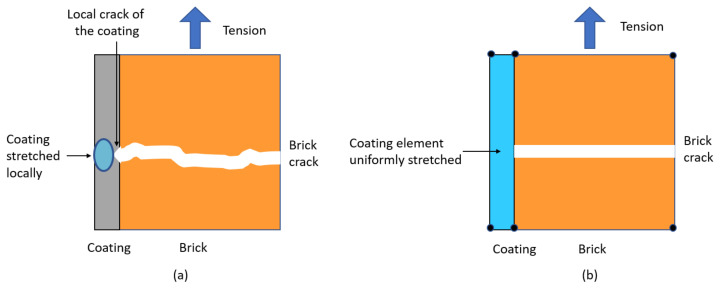
Comparison of the coating behaviour close to a crack in the experiment (**a**) and in the simulation (**b**).

The abovementioned findings highlighted that the reinforcement effects of both types of coating were difficult to model numerically. One reason could lie in the difference between the local stretching of the coating close to the crack that occurred during the experiment and the smoother elongation of the whole coating element in the simulation (Figure 11). The perfect adhesion [31,32,33,34] or the mesh densification of the retrofitting material [35,36] may not be totally sufficient to model its experimental contribution.

In order to address this limitation, a sensitivity study of the relevant parameters was performed. A similar study undertaken for URM walls of the same nature was presented in [30] and indicated that the Young’s moduli of the brick and of the joint had significant influences on the behaviour, while the tensile strengths of the joints and bricks, the Drucker Prager coefficient, and the characteristic strain of the joints played secondary roles. The present analysis focused on the parameters of the coating. It was also restricted to the wall with the MGF coating, as no significant difference was noticeable between the URM wall and the ISO-coated wall. Furthermore, the effect of the coating was expected to mainly operate under tension, when cracks occur in the bricks. Therefore, the study was restricted to the Young’s modulus E, the tensile strength $R_{T}$, the strain at the tension peak $\varepsilon_{PT},$ and the fracture energy under tension $G_{FT}$ (Table 4). A conservative value CV and an amplified value AV were tested for these parameters, except for the strain at the tension peak, which was already fixed to its minimal value in the reference case. For both the Young’s modulus and the tensile strength, the amplified value and the reference value were established by applying ratios of 1/3 and 3, respectively, to the value of the reference case. The same coefficient was used for the amplified value of the strain at the tension peak. Furthermore, a coefficient of 0.5 was used for the fracture energy under tension in the conservative case, which corresponded to brittle elastic behaviour. A coefficient of 10 was used for the fracture energy under tension in the amplified case, which is close to perfectly plastic elastic behaviour. 

The relative difference $\Delta_{RC}$ compared to the reference case RC was calculated in percent using the following equation (Equation (6)):

(6) $$\Delta_{RC}=100\times \frac{\left|CV-AV\right|}{RC}$$

The exception was the strain at the tension peak, for which $\Delta_{RC}$ was calculated as follows (Equation (7)):

(7) $$\Delta_{RC}=100\times \frac{\left|RC-AV\right|}{RC}$$

**Figure 12:**
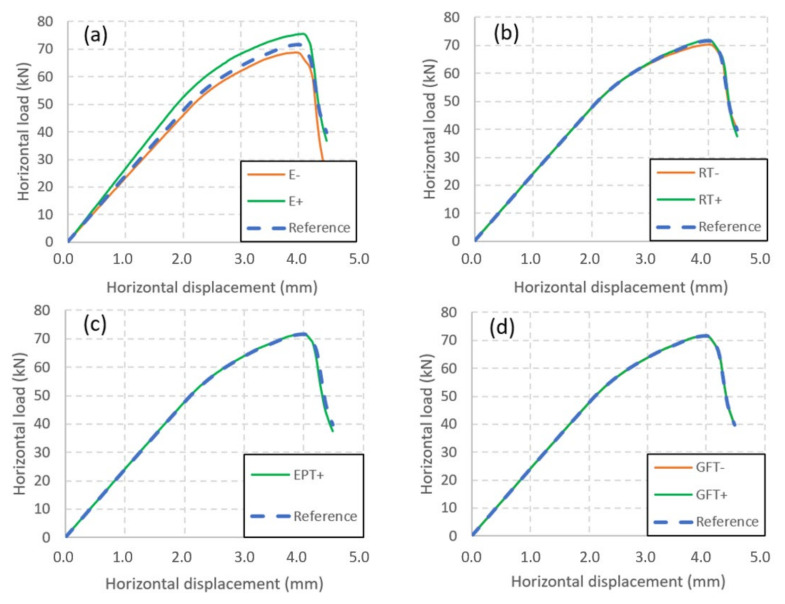
Influence of the coating parameters on the force–displacement curve of the masonry shear wall: (**a**) Young’s modulus; (**b**) tensile strength; (**c**) peak tensile strain; (**d**) fracture energy under tension.

Figure 12 and Table 5 summarize the results of the sensitivity study. The results show that the most sensitive parameter was the Young’s modulus, as has already been noticed [30]. Thus, the correct estimation of this parameter is of the utmost importance for the estimation of the bearing capacity of a coated wall. The influence of the tensile strength was also significant, but the gain offered by the amplified value was marginal. This aspect could be associated with the influence of the fracture energy under tension, which was almost null. This was also confirmed by another simulation in which a very high tensile strength was used, without generating any improvement in the bearing capacity. As a consequence, an improvement of the modelling approach is required to capture the effect of the coating correctly. This aspect should be considered when reinforcing materials that exhibit great deformability compared to the support they reinforce. 

### 3.4. Influence of the Vertical Loading Mode

As in the current study, in the pushover tests conducted in previous studies in the literature, a vertical load is applied to consider the effects of the dead and live loads on the specimen. The corresponding load is usually applied after the setup of the reinforcement. This makes a difference when the specimen is applied in a building, where the vertical loading already acts before the reinforcement is carried out. Therefore, two modes of the vertical loading configuration can be considered depending on whether a retrofitting or repair study is undertaken. In the following, the usual vertical loading mode is referred to as usual vertical loading (UVL). The second sequence is referred to as in-situ loading (ISL). This mode is not commonly used because of the complexity of sustaining the vertical load during the 28-day curing phase, as in [30]. Nevertheless, it corresponds to the in-situ setup of real walls, for which the coating is applied when the vertical is already present. This mode may produce different results from the UVL configuration. In particular, the part of the vertical load transmitted to the masonry is smaller in the UVL configuration than in the ISL configuration. The behaviour and the failure mode can also be affected by this aspect. This can occur with masonry coatings as well as with TRM or FRP reinforcements. Several configurations of materials were tested, ranging from a configuration for the MGF properties to one similar to the properties of TRM. For the sake of simplicity, only the Young’s modulus and the tensile and compression strengths were varied with the same multiplier coefficient ρ, while other parameters were defined as in Table 3. This coefficients were set to 1, 5, and 10, so that values similar to the properties of TRM [33,34,37,38] could also be assessed. Perfect adhesion was still assumed here, although this may not have been completely suitable to model a TRM-like material. An interface between the masonry and the reinforcement could have been incorporated to represent the sliding and debonding capacity [39]. For the sake of simplicity, this was not considered here. The three corresponding cases were denoted “MGF”, “intermediate”, and “TRM”, respectively (Table 6). As shown in Section 3.2, the effect of the ISO material was not retrieved by the current model. Hence, it was not considered here. 

**Figure 13:**
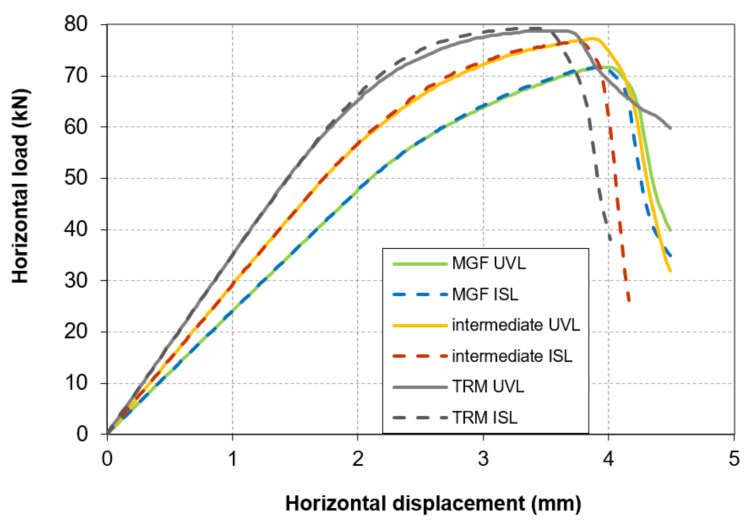
Influence of the in-situ stress: force vs. displacement curves for the tested loading configurations.

Table 6 indicates the vertical stress $\sigma_{V\_m}$ transmitted to the masonry after the application of the vertical loading. The variation for the three ISL configurations was marginal and can be explained by the low but not null value of the hydration coefficient used for the coating during the vertical loading phase. On the other hand, this vertical stress $\sigma_{V\_m}$ dropped by 36% in the MGF UVL configuration compared to the TRM UVL one. As a consequence, a significant part of the vertical loading was transferred by the reinforcement in this last case, and thus the failure mode could be modified [40]. From Table 6, it can be deduced that the effect of the vertical loading mode on the maximal strength was less than 1% (Figure 13). The post-peak behaviour was rather more dependent on the vertical loading configuration. It was even more significant for the TRM case. In the three cases tested, the strength dropped faster after the peak for the ISL mode than for the UVL loading mode.

**Figure 14:**
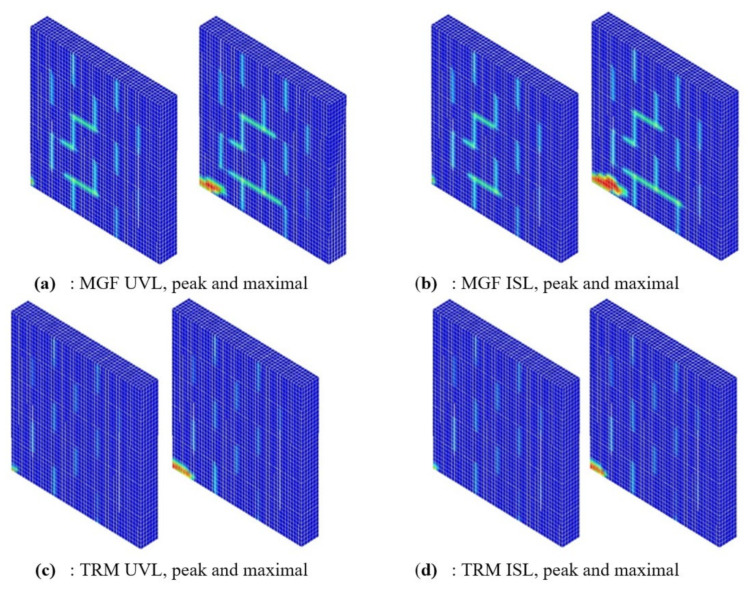
Compressive damage at peak and maximal displacement for the (**a**) MGF UVL, (**b**) MGF ISL, (**c**) TRM UVL, and (**d**) TRM ISL configurations.

Let us consider the pattern of damage under compression (Figure 14). As already shown in Figure 10, this can be an interesting index of staircase failure in masonry walls. A cross-comparison was undertaken for the type of vertical loading (UVL vs. ISL) and the type of reinforcement (MFG vs. TRM) and for both the maximal strength and the maximal displacement states. Damage under compression at the peak was quite independent of the loading mode for both the MGF and the TRM reinforcement. The difference between the two loading modes at the maximal displacement was also not significant for either of the materials presented. As a conclusion, we found that, even if the results were quite similar in term of strength and failure patterns, the limitations of the modelling approach seemed to annihilate the effect of the effective vertical stress applied to the masonry. Further investigations are required to establish the possible effect of the mode of vertical loading.

## 4. Conclusions and Perspectives

Pushover tests were performed for a series of masonry walls. Two of the walls were URM-like and were considered as reference cases. Two others were reinforced with a finishing coating, while an insulation coating was applied to the last two walls tested. The experiments were modelled with a finite element approach in order to analyse the results. Based on the results obtained, several statements can be made.

The experiments indicated that the coated walls exhibited higher shear strength than the reference walls. Furthermore, the reference walls underwent brittle failure while ductile failure characterized the coated walls. Even with low mechanical properties, the insulating coating enabled the reinforcement of the masonry. This could be considered in the future as a method to retrofit masonry buildings while undertaking refurbishment. 

A finite element model was developed to analyse the results. The reference walls were reproduced adequately in terms of strength and crack pattern evolution. The initial damage during the loading corresponded to the occurrence of a flexural failure at the base of the masonry. However, the primary behaviour was characterized more by staircase failure and corner crushing, which were clearly identified by the damage under compression variable $D_{C}$ in the model. This variable can be considered a relevant index of the state of masonry walls under shear loading. 

As observed during the experiments, the model of the wall covered with the MGF coating indicated an increase in strength compared to that for the URM one. On the other hand, no significant difference was noticed between the models of the URM wall and that with the ISO coating. This statement concerns both the maximal strength as well as the ductility behaviour. It can be assumed that the stress and strain concentration that occurred along the masonry cracks during the experiments was the main cause of this difference. Indeed, the localization of this elongation could not be retrieved by the model of the coating, the elongation being smoothed across the whole coating element. The model was almost insensitive to the tensile strength and the fracture energy under tension, highlighting the limits of the numerical approach. These features should be observed as long as the elastic strain of a reinforcement material is greater than that of the masonry. 

Two application modes of the vertical loading were compared, involving the setup of the coating before (ISL) and after (UVL) the application of the vertical loading. The results were quite similar in terms of maximal strengths and failure modes. On the other hand, the post-peak behaviour was dependent on the application mode, with a more brittle behaviour demonstrated when the in-situ loading (ISL) mode was considered. This last point should be considered in further experimental and numerical work for two reasons. First, this application mode is more realistic than the usual one. Furthermore, it was shown in Section 3.2 that the numerical model could not retrieve the experimental post-peak and ductile behaviours correctly. Hence, the difference in behaviour between the two loading mode configurations (ISL and UVL) could be more significant during an experimental analysis.

After overcoming these issues, the modelling of an entire construction should be carried out. Such an approach could be carried out with macro-elements [39,41] that incorporate the masonry and the presence of a coating. The gain in terms of bearing capacity offered by the coating for several loading configurations, including seismic loading, should be analysed. 

## Figures and Tables

**Table 1:** Mechanical properties of the tested coatings.

Coatings	E (MPa)	$\sigma_{t}\;\left(kPa\right)$	$\sigma_{c}\;\left(kPa\right)$
ISO	4 ± 1.4	70 ± 14	110 ± 30
MGF	300 ± 150	1289 ± 40	2900 *

* Mechanical property provided by the producer in the technical datasheet.

**Table 2:** Summary of wall test specimens.

Reference	Type of Coating	Coating Thickness (mm)
B1, B2	Uncoated	-
B3, B4	MGF	10
B6, B7	ISO	40

**Table 3:** Parameters set for the masonry and the coatings.

Materials	Brick	Vertical Joints	Horizontal Joints	Bed Joints	ISO Coating	MGF Coating
Young’s modulus E (MPa)	900		500	1000	4	600
Poisson ratio ν (-)	0.2					
Tensile strength $R_{T}$ (MPa)	0.5	10^−6^	0.1	0.1	0.07	1.29
Strain at tension peak $\varepsilon_{PT}$ (-)	1.0×RtE					
Fracture energy under tension $G_{FT}$ (MJ/m^2^)	$1.0\times \varepsilon_{t}\times R_{t}$					
Compression strength $R_{C}$ (MPa)	3.0	2 × 10^−6^	1.0	3.0	0.11	2.9
Strain at compression peak $\varepsilon_{PC}$ (-)	1.0×RcE					
Characteristic strain $\varepsilon_{k;s}$ (-)	5 × 10^−5^	10^−5^	3 × 10^−7^	3 × 10^−7^	10^−3^	10^−3^
Drucker Prager coefficient δ (-)	0.6	1.0	1.0	1.0	1.2	1.2
Dilatancy β (-)	5.0 × 10^−2^	10^−3^				
Reclosure characteristic stress $R_{R}$ (MPa)	$2.0\times R_{t}$					
Crack reclosure energy $G_{FR}$	$1.0\times G_{ft}$					

**Table 4:** Parameters used for the sensitivity analysis.

Parameter	Reference Value	Conservative Value (CV)	Amplified Value (AV)
Young’s modulus *E* (MPa)	600	200	1800
Tensile strength $R_{T}$ (MPa)	1.29	0.43	3.87
Strain at tension peak $\varepsilon_{PT}$ (-)	1.0×RtE	-	3.0×RtE
Fracture energy under tension $G_{FT,HJ}$ (MJ/m^2^)	$1.0\times \varepsilon_{t}\times R_{t}$	$0.5\times \varepsilon_{t}\times R_{t}$	$10\times \varepsilon_{t}\times R_{t}$

**Table 5:** Results of the sensitivity analysis.

Parameter	E	$R_{T}$	$\varepsilon_{PT}$	$G_{FT}$
Conservative value CV	68.67	70.32	-	71.73
Amplified value AV	75.39	72	71.55	71.73
Difference from reference case $\Delta_{RC}$ (%)	9.4	2.3	0.2	0

**Table 6:** Properties of the three configurations tested and main results.

Name	Loading Type	Coefficient ρ (-)	E $\left(MPa\right)$	$R_{T}$ $\left(MPa\right)$	$R_{C}$ $\left(MPa\right)$	$\sigma_{V_{m}}$ $\left(MPa\right)$	$F_{H\_max}$ $\left(kN\right)$
MGF	UVL	1.0	600	1.29	2.9	0.65	71.73
	ISL					0.691	71.67
Intermediate	UVL	5.0	300	6.45	14.5	0.517	77.19
	ISL					0.691	76.68
TRM	UVL	10.0	6000	12.9	29.0	0.415	78.80
	ISL					0.689	79.23

## Data Availability

The data presented in this study are available upon request from the corresponding author.
